# The relationship between physical activity and prosocial behavior of college students: A mediating role of self-perception

**DOI:** 10.1371/journal.pone.0271759

**Published:** 2022-08-05

**Authors:** Tian Ci Lu, Cai Xia Wang, Bao Le Tao, Hao Ran Sui, Jun Yan

**Affiliations:** College of Physical Education, Yangzhou University, Yangzhou, Jiangsu, China; Qatar University College of Education, QATAR

## Abstract

**Objective:**

To explore the relationship between physical activity and prosocial behavior in college students, and to examine whether self-perception and gender may play mediating and moderating roles, respectively, in that relationship.

**Methods:**

The International Physical Activity Questionnaire-long form, Prosocial Tendencies Measure, and Self-perception Scale were used to survey 647 college students in Yangzhou, China. Internal consistency testing, one-way analyses of variance (ANOVAs) across physical activity levels, exploratory factor analysis, correlation testing, mediation effect testing (independent variable, physical activity; mediating variable, self-perception; dependent variable, prosocial behavior), bootstrap testing and moderated mediation testing were conducted.

**Results:**

Physical activity level was not found to be a direct predictor prosocial behavior in college students. Self-perception was found to play a mediating role between physical activity and prosocial behavior.

**Conclusion:**

Physical activity is not directly predictive college students’ prosocial behavior tendencies, but it is indirectly predictive through self-perception. This study explores the relationship between the three variables and the path of the relationship, deepening the research related to the relationship between physical activity and prosocial behavior, providing ideas for fostering prosocial behavior in Chinese universities, as well as providing a theoretical basis for possible future empirical research.

## Introduction

Prosocial behavior is defined as behavior that individuals engage in during social interactions and in their relationships that conform to social norms while being beneficial to others and society [1]. It can facilitate adaptation of individuals to a society and its presence is an important sign of the development of socialization in individuals [2]. In college settings, it can reduce interpersonal tensions and enable students to perceive more social support while helping students to adapt to academic and life pressures and helping students to better cope with anxiety and depression caused by social competition, confusion, and other negative emotions [3].

Anti-social behaviors, which emerge most commonly in adolescence, can be associated with biological and environmental factors in most cases. In recent years, there have been numerous incidents on college campuses with obvious anti-social characteristics, such as cruelty to animals and deliberate destruction of dormitory housing. Without appropriate intervention, adolescent anti-social behaviors may develop into lifelong societally destructive behavioral patterns [4].

Therefore, research on prosocial behavior can effectively prevent Chinese university students from developing anti-social behavior problems, and can provide methods and ideas for cultivating the physical and mental health of students in Chinese universities.

Physical activity has been shown to promote the development of prosocial behaviors in adolescence, in large part by improving self-efficacy and one’s ability to deal with negative emotions by cultivating sociability personality traits [5]. Compared to their sedentary peers, highly active adolescents have been shown to have significantly lower levels of interpersonal anxiety together with significantly higher self-elasticity and prosocial behaviors [6]. Therefore, in the present study, we examined the following first research hypothesis: college students’ physical activity can benefit prosocial behavior directly.

Self-perception refers to multi-level knowledge of oneself, including being cognizant of one’s own appearance, bodily internal states, and mental activities [7]. It has been suggested that increasing one’s self-perception may lead one to reflect on their own values and ideals, and such reflections may promote prosocial behaviors [8]. Some studies have shown that highly active college students self-report having better views of themselves than students with a low level of physical activity [9–12]. Some studies have found that physical activity led adolescents to have a better self-concept, which improved their confidence in establishing good interpersonal relationships with peers and teachers. Confidence can facilitate the formation of an attitude that favors experiencing society positively, which promotes the formation and development of prosocial behaviors in adolescents [13]. Shavelson considers self-concept as a hierarchical and multidimensional category construct [14], while Liao et al.’s study indicates that Chinese university students’ self-concept is more concerned with the evaluation and perception of self [15], social cognitive theory argues that an individual’s perception of self and assessment of self-efficacy can influence their social behavior, such as prosocial behavior [16]. And life-course and personality development theories also suggest that adolescents are in the midst of self-identity and role confusion conflicts, a stage that may lead them to develop the capacity for empathy and thus correlate with the development of prosocial behavior [17].

Therefore, self-perception may have a very crucial role in the enhancement of the level of prosocial behavior. And in the study of the relationship between physical activity and prosocial behavior among Chinese university students, its’ relevant to explore the mechanisms of self-perception in the relationship.

Based on these findings, the following second research hypothesis was examined in this study: physical activity can affect prosocial behaviors indirectly through self-perception as an intermediary variable.

The current relationship between physical activity, self-perception and prosocial behavior has not yet been discussed, exploration of the relationship between the three variables can lead to a deeper study of the mechanisms underlying the relationship between physical activity and prosocial behavior. Therefore, this study will take this as a research direction, hoping to provide new ideas for the development of prosocial behavior of university students and provide a theoretical basis for possible future empirical studies.

**Fig 1:**
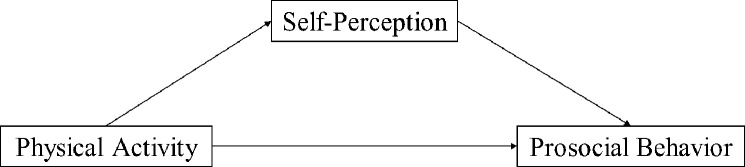
Theoretical model diagram.

Hence, this research proposes and tests a model of the relationship between physical activity and prosocial behaviors, wherein physical activity is a positive predictor of prosocial behavior and self-perception plays an intermediary role between these two entities (Fig 1).

## Materials & methods

### Ethical approval

The study was carried out ethically, and was approved by the Ethical Committee of Yangzhou University Medical college (No. YXYLL-2021-146).

### Sample

University students (19-22years old) in Yangzhou city were reached through the Questionnaire Star distribution platform (Questionnaire link shared by teachers). All questionnaires are completed online, participants are voluntary and can withdraw from the questionnaire at any time The questionnaires were downloaded and collated by researcher. The group sampling method was used to recruit 646 students, all of whom returned their questionnaires (100% recovery). Following the exclusion of irregular and incomplete questionnaires, 532 questionnaires were obtained (82% efficiency). The final sample of 532 students included 207 man (39%) and 325 woman (61%).

### Research tools

#### Prosocial tendencies measure

The original PTM was designed by Carlo and Randall [18]. We employed a revised Simplified Chinese version of the PTM developed by Kou and colleagues [19, 20].It has 26 questions across six dimensions: emotional, obedient, altruistic, anonymous, public, and urgent. Each item was answered on a 5-point Likert scale, with higher scores indicating more prosocial behaviors. The Cronbach’s α coefficient of the PTM in this study was 0.962.

#### Self-perception scale

Zhang’s [21] modified Self-perception Scale, based on the Adolescent Mental Health Quality Questionnaire compiled by Jiang, was employed. It has 8 questions across three dimensions: physical self-perception, academic self-perception, and emotional self-perception. The items were answered via a Likert 4-point scoring method. The Cronbach’s α coefficient for this scale in this study was 0.8.

#### International Physical Activity Questionnaire-long form (IPAQ-L)

The IPAQ-L questionnaire was compiled by the International Physical Activity Measurement Working Group [22], and was translated into Chinese by Qu and Li [23]. The questionnaire consists of five sections: occupation, housework, transportation, leisure, and meditation. This questionnaire has been widely used in physical activity surveys among Chinese university students [24], and the Chinese version of the IPAQ has shown high reliability and validity [23], The Cronbach’s α coefficient of the IPAQ-L in this study was 0.6.

### Data processing

Calculate the MET score by multiplying the MET value corresponding to each activity in the IPAQ questionnaire by the time (in minutes) and frequency (in days) to obtain a weekly sum of physical activity levels. The scores of the prosocial tendencies measure and the self-perception scale were also summed up separately, and finally the scores of the three questionnaires were imported into SPSS software for processing. Internal consistency testing, one-way analyses of variance (ANOVAs) across three physical activity level groups (low, medium, and high), exploratory factor analysis, and correlation testing were conducted in SPSS, version 26.0 (IBM, USA). Mediation effect testing, bootstrap testing and moderated mediation testing, were conducted with the Process macro contributed by Hayes [25], version 3.3, in SPSS. For mediation effect testing, a simple mediation model (Model 4) was developed with physical activity as an independent variable, self-perception as a mediating variable, and prosocial behavior as a dependent variable. The criterion for statistical significance was *p* < 0.05.

## Results

### Descriptive and correlational analyses of the study variables

The mean physical activity levels of man students were significantly higher than those of woman students, while self-perception scores and prosocial behavior scores were found to be similar between man and woman (Table 1). One-way ANOVAs indicated that physical activity did not have significant main effects on self-perception or prosocial behavior (Table 2). Correlation analyses (Table 3) showed that students’ physical activity levels correlated positively with their prosocial behavior scores and self-perception scores. Additionally, self-perception scores correlated positively with prosocial behavior scores (Table 3). These correlation analysis results provided a basis for further exploration of a mediating effect.

**Table 1:** Comparison of study variables between man and woman.

Variable	Mean level/score ± SD by gender group		t	*p*	Cohen’s d
	Man (N = 207)	Woman (N = 325)			
Physical activity	1782.36 ± 2592.88	1379.07 ± 1458.34	2.04	**0.042**	0.19
Prosocial behavior	100.01 ± 19.43	99.34 ± 14.42	0.49	0.628	0.04
Self-perception	22.59 ± 4.48	22.29 ± 4.01	0.82	0.414	1.13

SD, standard deviation.

**Table 2:** One-way ANOVAs of physical activity level effects on self-perception and prosocial behavior scores.

Variable	Mean score ± SD by physical activity level group			F	*p*
	High (N = 121)	Medium (N = 242)	Low (N = 169)		
Prosocial behavior	100.35 ± 15.81	100.26 ± 14.85	98.22 ± 19.14	0.91	0.41
Self-perception	22.96 ± 4.07	22.36 ± 3.83	22.08 ± 4.73	1.57	0.21

SD, standard deviation.

**Table 3:** Summary of correlation coefficients among the study variables.

Variable	Mean	SD	1	2	3	4
1 Gender	0.61	0.49	–	–	–	–
2 Physical activity	1535.99	1968.10	-0.099*	–	–	–
3 Prosocial behavior	99.63	16.53	-0.022	0.088*	–	–
4 Self-perception	22.41	4.20	-0.035	0.117**	0.452**	–

*p < 0.05, *p < 0.01. SD, standard deviation.

### Mediation effect

The β, t, R^2^, and F values obtained from mediation effect testing with 5000 samplings obtained by the Bootstrap method are reported in Table 4. Note that the total effect regression equation was confirmed to be highly significant. Although physical activity did not have a predictive effect on prosocial behavior (*p* = 0.36), self-perception was a significant positive predictor of prosocial behavior (*p* < 0.001) and physical activity was a significant positive predictor of self-perception (*p* = 0.007).

**Table 4:** Mediation effect testing results (standardized).

Variable relation	β	SE	t	*R* ^*2*^	F
Physical activity prediction of self-perception	0.1170	0.431	2.7121**	0.0137	7.3558**
Physical activity prediction of prosocial behavior	0.358	0.390	0.9165	0.2059	68.5830**
Self-perception prediction of prosocial behavior	0.4482	0.390	11.4882**		
Regression of physical activity total effects	0.0882	0.0433	2.0383	0.0078	4.1546**

*p < 0.05

**p < 0.01. SE, standard error.

Effect size values are reported in Table 5. Note that analysis of Bootstrap 95% confidence intervals affirmed a significant mediating effect of self-perception on self-perception (i.e., 0.05424). Thus, although physical activity may not predict prosocial behavior directly, it can predict prosocial behavior indirectly through a mediating effect of self-perception. This direct effect and mediating effect accounted for 40.6% and 59.4% of the total effect of physical activity (i.e., 0.0882), respectively. These data do not confirm hypothesis 1 but do confirm hypothesis 2 by demonstrating complete mediation.

**Table 5:** Total, direct, and mediating effects.

Effect	Effect	BootSE	Boot LLCI	BootULCI	Relative effect ratio
Total effect	0.0882	0.0433	0.0420	0.0032	–
Direct effect	0.0358	0.0365	-0.0406	0.1019	40.6%
Mediating effect of self-perception	0.0524	0.0218	0.0092	0.0960	59.4%

BootSE, Bootstrap standard error; BootLLCI, Bootstrap lower limit of the 95% confidence interval; BootULCI, Bootstrap upper limit of the 95% confidence interval.

## Discussion

### Relationship between physical activity and prosocial behavior

College is a secondary socialization setting where people undergo a transition from late childhood to adulthood [26]. Cultivation of prosocial behavior is closely related to the future development of a healthy society. Studies have shown that individuals with high prosocial behavior tendencies have more interpersonal relationships than individuals with low prosocial behavior tendencies [27, 28]. Our first hypothesis that physical activity can have a direct positive effect on prosocial behavior was not confirmed. Putra and colleagues found in a study in which physical activity was a mediating variable that the mediating effect of physical activity was not significant between green space quality and prosocial behaviors [29]. That conclusion is consistent with our finding in showing that physical activity did not predict prosocial behavior directly. Likewise, according to a one-way ANOVA, prosocial behavior scores did not differ significantly among college students with high, moderate, and low levels of physical activity. It is possible that a subtle effect that could not be detected with our use of the IPAQ-L questionnaire, which summarizes total physical activity over an extended period of time, might become evident and significant with a more targeted instrument.

College students can gain exposure to physical activity through social and work-study activities. Since the 1980s, Chinese college campuses have been affected by the Western concept of work-study. With the recent rapid development of Chinese society, the motivation for obtaining part-time jobs among college students has changed from relieving economic pressure to increasing social experiences and acquiring contacts. However, most college students’ jobs are in low-skilled labor roles [30].

Regular and specialized exercise with adequate intensity can benefit physical and mental health [31, 32]. Hence, our inability to confirm self-reported physical activity level data with IPAQ-L responses is a limitation of this study. Moore showed that moderate-intensity physical activity correlated with prosocial behavior, while neither low- nor high-intensity physical activity did [33]. Therefore, it may be explicatory to explore the effects of physical activity on prosocial behavior in college students in studies in which there is teacher-directed regular and moderate-intensity physical activity.

### The mediating role of self-perception

Physical activity was confirmed to be a positive predictor of self-perception, such that higher levels of physical activity tend to lead to better self-perception, with a corresponding improvement in prosocial behavior, consistent with previous studies [11, 34]. Previously, physical activity has been positively associated with the physical and mental health of college students, affirming the notion that engaging in regular physical activity is a healthful practice. Individuals who do so report perceiving positive changes in themselves, thereby obtaining continuous positive feedback that leads to enhanced self-efficacy, self-affirmation, and self-confidence levels [35, 36]. This virtuous circle can increase psychological capital, reduce one’s sense of inferiority, improve self-confidence, and help young adults form positive and healthy values, world views, and other characteristics related to the formation and development of self-perception, thus leading to good self-perception. Indeed, higher levels of physical activity have been shown to lead to greater self-perception [37].

Self-perception can be a positive predictor of prosocial behaviors. Erikson’s theory of life-course and personality development posits that adolescents experience a conflict of self-identity and role confusion, in which they are hyper-attentive to their own image in the eyes of others and their socio-emotional position among their peers and community. In the process, one develops the ability to distinguish between the self and others as well as the ability to empathize with others to some extent [38, 39]. Individuals who have developed these abilities show prosocial behaviors such as caring for others, sharing, giving, and compassion [40]. Accordingly, those with a high self-perception ability tend to exhibit an ability to understand themselves more clearly, stably, deeply, and accurately; and they exhibit prosocial behaviors simultaneously. Conversely, those with a low self-perception ability may be more superficial and one-sided in their cognition and self-evaluation. That disposition tends to have a negative impact on interpersonal interactions, which in turn undermines one’s confidence in their ability to build good interpersonal relationships. Individuals with low social confidence are less likely to seek positive social experiences, which ultimately has adverse effects on the development of prosocial behavior. This view is also supported by social concept theory, which states that individuals assess their ability to perform prosocial behaviors or the effects on themselves and others if they do so [16]. Thus, personality development and social cognitive theories appear to be complementary, and together they can explain the positive relationship between self-perception and prosocial behavior.

## Conclusions

The current research provides information on the relationship between physical activity and better prosocial behavior in college students, as well as the mediating role of self-perception in that relationship. Though physical activity did not predict prosocial behavior directly, self-perception was shown to play a complete mediating role between physical activity and prosocial behaviors in college students.

This finding expands some ideas for research related to the relationship between physical activity and prosocial behavior. i.e., physical activity may be related to an individual’s prosocial behavior through self-perception.

## Limitations and future research

This article examines the relationship between physical activity, self-perception and pro-social behavior among university students. As this study used a questionnaire research method, all questionnaires were self-assessed by the subjects, which has the problem of lack of objectivity. The IPAQ questionnaire also collects information on the physical activity of individuals in all four areas of daily work, daily transportation, daily life and sports and exercise in a week, which may also have the problem of being unobjective and irregular.

In future research, in order to address the subjective bias caused by the questionnaire and to explore the causal relationship between physical activity and prosocial behavior by using experimental methods of motor intervention to maximize control for confounding items. Meanwhile, further expansion of the survey sample will be considered to examine whether the results are influenced by cultural differences, economic differences, and differences in educational settings across geographic regions.

## Supporting information

S1 File RAW data. (XLSX)

S2 File RAW data SPSS. (SAV)
